# Combination of Nanomaterials in Cell-Based Drug Delivery Systems for Cancer Treatment

**DOI:** 10.3390/pharmaceutics13111888

**Published:** 2021-11-08

**Authors:** Lu Tang, Shun He, Yue Yin, Hening Liu, Jingyi Hu, Jie Cheng, Wei Wang

**Affiliations:** 1 State Key Laboratory of Natural Medicines, Department of Pharmaceutics, School of Pharmacy, China Pharmaceutical University, Nanjing 211198, China; lutang@stu.cpu.edu.cn (L.T.); heshun@stu.cpu.edu.cn (S.H.); yinyue@stu.cpu.edu.cn (Y.Y.); liuhening@stu.cpu.edu.cn (H.L.); hjy@stu.cpu.edu.cn (J.H.); 2 NMPA Key Laboratory for Research and Evaluation of Pharmaceutical Preparations and Excipients, China Pharmaceutical University, Nanjing 211198, China; 3 Jiangsu Key Laboratory of Oral Diseases, Nanjing Medical University, Nanjing 210029, China; 4 Department of Oral and Maxillofacial Surgery, Affiliated Hospital of Stomatology, Nanjing Medical University, Nanjing 211166, China

**Keywords:** cell therapy, biomimetic strategy, nanomaterial, cancer treatment, targeted drug delivery

## Abstract

Cell-based drug delivery systems have shown tremendous advantages in cancer treatment due to their distinctive properties. For instance, delivery of therapeutics using tumor-tropic cells like neutrophils, lymphocytes and mesenchymal stem cells can achieve specific tumor targeting due to the “Trojan Horse” effect. Other circulatory cells like erythrocytes and platelets can greatly improve the circulation time of nanoparticles due to their innate long circulation property. Adipocytes, especially cancer-associated adipocytes, play key roles in tumor development and metabolism, therefore, adipocytes are regarded as promising bio-derived nanoplatforms for anticancer targeted drug delivery. Nanomaterials are important participants in cell-based drug delivery because of their unique physicochemical characteristics. Therefore, the integration of various nanomaterials with different cell types will endow the constructed delivery systems with many attractive properties due to the merits of both. In this review, a number of strategies based on nanomaterial-involved cell-mediated drug delivery systems for cancer treatment will be summarized. This review discusses how nanomaterials can be a benefit to cell-based therapies and how cell-derived carriers overcome the limitations of nanomaterials, which highlights recent advancements and specific biomedical applications based on nanomaterial-mediated, cell-based drug delivery systems.

## 1. Introduction

Recent decades have witnessed the tremendous progress of nanobiotechnology in cancer treatment [1,2]. Nano-based drug delivery systems (DDS) are one of the most widely investigated strategies to improve the targetability of therapeutic molecules, increase circulation time, and enhance the total bioavailability [3]. As important constituents of nano-based DDS, various nanomaterials like organic nanomaterials, inorganic nanomaterials, and hybrid nanomaterials have been intensively explored in anticancer drug delivery, owing to their unique properties [4,5,6]. The delivery of therapeutic agents using nanomaterials holds numerous advantages over their free drug counterparts, which can not only protect the encapsulated drugs from degradation or inactivation before reaching sites of action, but also enable the controlled drug release in specific environments. In addition, both passive and active targeting can be achieved via the enhanced permeability and retention (EPR) phenomenon, or by means of extra modification [7,8]. Moreover, owing to the EPR effect, nanosystems possess the ability to improve the accumulation of chemotherapeutics both as single agent and in combination, which largely elevates the amounts of drugs in target tissue [9,10,11,12]. When it comes to active targeting, two main approaches are currently adopted to improve tumor accumulation of nanoparticles. One is to apply targeting molecules to endow the nanosystems with targetability [13,14]. The other is to modulate the protein corona of nanocarriers to provide a “natural targeting” towards TME [15,16,17]. In theory, nano-based DDS can be employed as an ideal vehicle in cancer treatment. However, many obstacles still impede the wide application of nanomedicine. For instance, although the EPR effect and active targeting approaches can modulate the biodistribution of nanomedicine to a certain extent, only a part of nanomedicine can reach the tumor sites while the majority of them are cleared by the reticuloendothelial system (RES) [18]. Moreover, EPR effect tends to be more efficient in some angiogenic tumor models with leaky blood, there are still some cases that are not suitable for EPR effect [19]. Besides, modification using targeting ligands could potentially compromise the stealth ability of the nano-based DDS [20]. Therefore, other novel strategies in combination with nanotechnology is of great necessity to achieve improved therapeutic performance.

**Figure 1:**
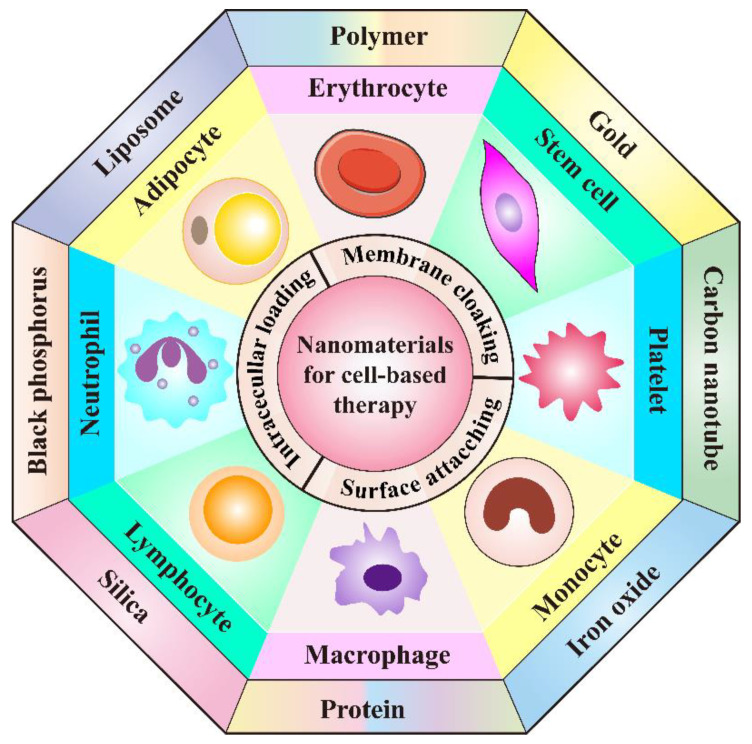
Schematic illustration of the cooperation of cell-based therapies with novel nanomaterials in cancer treatment.

Hopefully, cell-mediated drug delivery has become a promising approach in addressing the aforementioned limitations. This innovative strategy takes advantage of the natural properties of various cells such as prolonged circulation time in blood stream, specific targetability to tumor cells, the ability to cross challenging biological barriers, abundant surface ligands, flexible morphology, and cellular signaling or metabolism [21]. Very recently, d’Avanzo et al. reported a kind of peptide-functionalized-liposomes that were able to selectively bind breast cancer cells, which in vitro demonstrated the ability of the resultant liposomes to target M2-macrophages to exploit a potential hitchhiking effect in vivo, bridging the gap between conventional nanosystems and cell-derived ones [22]. Therefore, cooperating advanced nanomaterials with cell-based therapies can largely strengthen the total therapeutic efficacy through maximizing the advantages of both, while minimizing their inherent shortcomings. In this review, a number of novel DDSs, based on various cell types including leucocytes, erythrocytes, platelets, stem cells, and adipocytes will be highlighted (Figure 1). Different cell types possess distinctive properties, which enables their multifunctional application in personalized cancer treatment. It is discussed how nanomaterials empower the field of cell-based treatment and how cellular characteristics improve the performance of nanomedicine. Diverse delivery strategies that utilize living cell internalization or cell membrane-cloaking in cooperation with multiple treatment modalities including chemotherapy, phototherapy, gene therapy and immunotherapy will be introduced in detail. Table 1 is a summary of nanomaterials in cell-based drug delivery for cancer treatment. In addition, compared to the existing review articles about cell-based therapies, the novelty of this review is that we comprehensively summarize the most widely used cell types in cyto-pharmaceuticals, which highlights the combinational strategy of innovative nanomaterials and cell-derived vectors with very latest research examples [21,23]. Moreover, the current limitations and future orientations for cell-based therapies are also discussed in this paper to provide more detailed instruction for the development of cell therapies.

## 2. Leukocyte-Based Drug Delivery Systems

**Figure 2:**
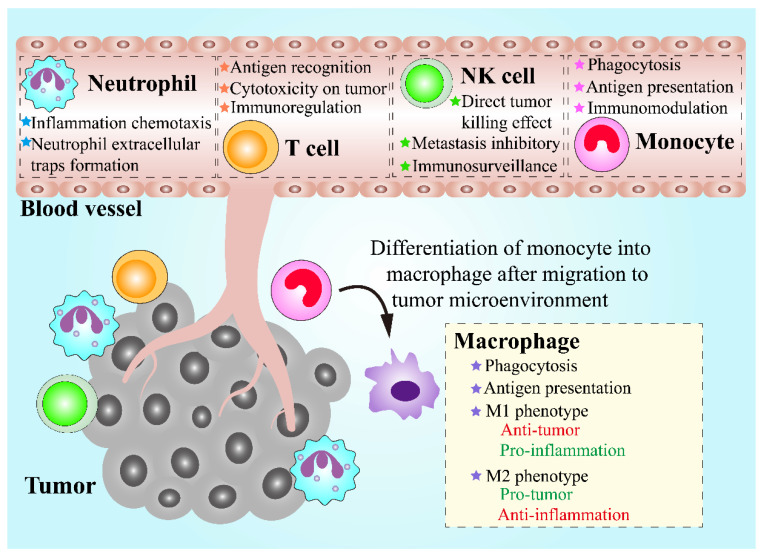
Schematic demonstration of the properties of various leukocytes and their functions in antitumor therapies.

Leukocytes, also called white blood cells (WBCs), are a group of colorless cells with a nucleus, in circulation, which are associated with innate and adaptive immune responses [57]. Circulating leukocytes can be mainly divided into five categories based on their morphological and bio-functional features, including neutrophils, monocytes, lymphocytes, eosinophils, and basophils [58]. Among the leukocytes groups, neutrophils, eosinophils, and basophils are collectively known as granulocytes, in which numerous granules are stored and can be released from the cytosol into extracellular space [59]. Chemotaxis is a fundamental ability of leukocytes, which means that leukocytes can migrate towards chemoattractants like formyl peptides, leukotrienes and complement fragments, or migrate away from chemorepellents [60]. When the infection, inflammation or cancer occurs, the recruitment of leukocytes can be triggered, leading to an increasing number of leukocytes in circulation and their follow-up transmigration [61]. Therefore, leukocytes are regarded as promising candidates for the implementation of the treatment of inflammatory diseases and cancers. During the tumor initiation and progression, numerous leukocytes are recruited towards tumor sites, which is known as leukocyte infiltration and can contribute to an inflammatory tumor microenvironment (TME) [62]. In addition to tumor-infiltration leukocytes that play a crucial role in TME, leukocytes in circulation also have great potential for cancer treatment via serving as vehicles. Leukocytes circulating in blood vessels are more likely to locate near the vessel wall instead of the vessel center, causing adhesive interactions between leukocytes in circulation and endothelial cells within the inner blood vessel through intercellular adhesion molecule-1 (ICAM-1) on endothelial cells [21,63]. Interestingly, circulating tumor cells that are essential to tumor progression and metastasis share similar migration and adhesion characteristics in blood vessels as leukocytes, in circulation [64]. Therefore, leukocytes can be potentially exploited as an ideal DDS for cancer treatment (Figure 2).

### 2.1. Neutrophils

Neutrophils are the most abundant leukocytes with unique nuclear morphology and defined granule content, which are a kind of myeloid cells that are sourced from immature neutrophil precursors in the bone marrow [65]. In addition, neutrophils play a crucial role in the immune response against infection and inflammation. Under infectious and inflammatory conditions, the life span of neutrophils is significantly extended, which allows neutrophils to persist for a sufficient period to express cytokines, release bioactive molecules such as granules, and mediate the recruitment of other immune cells [66]. More importantly, not only the release process of neutrophils from the bone marrow to circulation, but also the mobilization of neutrophils to inflammatory and tumor niches, are involved in neutrophil chemotaxis, that is mediated by chemokine receptors and integrin adhesion receptors expressed on neutrophils [67]. In addition to chemotaxis capacity, another important characteristic is neutrophil extracellular traps (NET) formation, whose process is also described as NETosis. NETosis is, in fact, a type of cell death of neutrophils which differs from neutrophil necrosis and apoptosis. In infection, inflammatory disorder niches and TME, NETosis occurs companied by release of intracellular contents, such as chromatin, granules of neutrophils, and carried cargoes [68]. Taken together, neutrophils have great potential to be an excellent drug delivery carrier with tumor targeting nature and drug release ability within TME for cancer therapy.

A variety of nanomaterials have been utilized in DDS in cooperation with neutrophils for cancer treatment. One approach to apply neutrophils as a drug delivery vector is to construct biomimetic drug loaded nanoparticles (NPs) with neutrophils membrane coating. PLGA, fully named as poly (lactic-co-glycolic acid), is a widely used polymer due to its high biocompatibility and biodegradability [69]. Moreover, formulation based on PLGA can achieve a sustained drug release behavior for periods up to 6 months, and up to 19 formulations involved in PLGA material have been approved by FDA [70]. Wang et al. developed PLGA-based NPs that were coated with a neutrophil membrane to carry the chemotherapeutic compound paclitaxel (PTX) for ovarian adenocarcinoma treatment [24]. PTX was preloaded in the PLGA core, then the core was encapsulated by the membrane of neutrophils which was further modified with a tumor necrosis factor-related apoptosis-inducing ligand (TRAIL). The developed system, named TNM-PN, displayed great serum stability and could release PTX in a sustained manner in vitro. Moreover, TRAIL on TNM-PN surface promoted the uptake of TNM-PN by the SKOV3 cells while the neutrophil membrane coating enabled TNM-PN to escape immune elimination as well as prolong their circulation in vivo. In vitro experiments showed that TNM-PN could selectively bind with inflamed human umbilical vein endothelial cells (HUVECs) and in vivo results demonstrated the specific biodistribution and high accumulation of TNM-PN at tumor sites, both indicating the excellent targeting capacity of the constructed nanocomplexes that is a product of the neutrophil membrane clocking. More crucially, SKOV3 tumor-bearing nude mice in TNM-PN treatment group showed significant tumor inhibition compared with other groups, confirming the excellent targeting capacity, successful drug release and high antitumor efficiency of TNM-PN.

Apart from the neutrophil membrane cloaking strategy, another approach to employ neutrophil in drug delivery is the hitchhiking strategy, which means that living neutrophils can pick up NPs and transport them. Based on this concept, Li et al. established a pathogen-mimicking NP, called nano-pathogenoids (NPNs), which could be recognized and internalized by neutrophils, due to the immune response to fight pathogen of neutrophils [25]. NPNs were in a core–shell structure and prepared from cisplatin and photothermal transducer polymer PBIBDF-BT, loaded PEG-b-PLGA mi-cellar NPs, encrusted with outer membrane vesicles (OMVs), which were secreted from Escherichia coli bacteria. Hence, NPNs with pathogenic features could be recognized and internalized by neutrophils in circulation. Additionally, neutrophils could retain the chemotaxis ability after the uptake of NPNs, for which NPNs were capable of hitchhiking neutrophils in circulation to arrive at inflamed issue. Upon photothermal therapy (PTT), numerous tumor cells were killed, accompanied by TME inflammation and follow-up neutrophils recruitment and inflation. Therefore, NPNs could be transferred and accumulated at inflamed tumor tissue as the in vivo results showed, displaying great tumor targeting ability after PTT. In vitro results also revealed that neutrophils could achieve NET formation to release carried contents when they were treated with phorbol myristate acetate mimicking inflammatory condition, and the re-leased NPNs and cisplatin were still active to cause EMT6 cell death. More crucially, in vivo results showed that, once the neutrophil containing NPNs reached the tumor niche, NPNs were sufficiently released and were taken up by EMT6 tumor cells. Finally, this PTT, in combination with neutrophil-mediated chemotherapy, contributed to a 60% tumor-free rate and a 97% tumor growth inhibition, which completely eradicated all tumors after repeated treatment in EMT6-bearing mice.

### 2.2. Lymphocytes

Lymphocytes are the second largest subtype of leukocytes in human, taking up about 30% of the population [71]. The main subsets of lymphocytes, including T lymphocytes (T cells), B lymphocytes (B cells), and natural killer cells (NK cells), have an essential effect on host immunity and perform their individual function against virus-es, bacteria, and tumor cells, due to their different roles in the immune system. For cancer treatment, lymphocytes or engineered lymphocytes have gained increasing concerns because of the rapid development of cancer immunotherapy [72]. T cell is a critical participant of the adaptive immune system in response to a broad array of antigens mediated by T cell receptors [73]. CD8^+^ T cells, known as cytotoxic T lymphocyte (CTL), and CD4^+^ T cells, also named helper T cells, are two important subsets of T cells, which are considered as the principal weapons of immunity against cancer [74]. T cells are capable of homing to the TME where tumor-associated antigens exist [75]. Unlike T cells, NK cells can directly kill cancer cells by secreting cytokines and perform their cytotoxic effect with no demand of the previous encounter with the antigen [76]. Moreover, NK cells are members of the innate immunity system and can recognize the target antigen, independently from major histocompatibility complex I (MHC-I). Based on NK cell receptors regulation, NK cells are capable of realizing the identification and targeting of cancer cells [77]. Moreover, both T cells and NK cells have been engineered to express chimeric antigen receptors (CARs) with tumor specificity, which promotes their efficacy and empower their functions [78]. Therefore, T cells and NK cells show great potential to mediate drug delivery for cancer therapy.

#### 2.2.1. T Lymphocytes

Drug transportation based on T cells have been investigated and several types of drug delivery platform have been proposed. One of the developing approaches of the T cell-based drug delivery strategy is to load drugs into T cells. Steinfeld et al. compared two normal loading methods of T cells’ incorporation with doxorubicin (DOX)-laden magnetite NPs, including electroporation and endocytosis. It was concluded that endocytosis by a smooth coincubation process was an effective method of drug uptake into cells, along with a reduced death rate of T cells [79]. In light of this, Kennedy et al. applied an endocytosis method by colocalization of gold nanospheres (AuNPs) to be loaded into T cells, which demonstrated a high loading efficiency as well as no influence on viability and function of T cells. Both in vitro and in vivo studies confirmed that AuNP-loaded T cells retained their homing ability to tumor sites and achieved specific tumor AuNPs accumulation, displaying the potential to promote the effect of AuNPs-based phototherapy [26].

Apart from loading therapeutic agents or NPs inside cells, attachment of NPs on the surface of T cells is an alternative approach to develop T cell-based drug delivery vehicles. Liposome is an ideal nanomaterial incorporated with T cells due to convenient functionalization with thiol-reactive group for covalent attachment to exofacial thiols on T cell surfaces, based on disulfide bond formation. Wayteck et al. constructed a siRNA-loaded liposome coupled to the surface of CTL [80]. The pyridyldithiopropionate (PDP)-functionalized lipids were incorporated in liposomes bilayer to form PDP-liposome that could couple with free thiol groups highly expressed on CTL mem-brane via a disulfide bond formation. The formed disulfide bond could respond to re-duction stress such as glutathione, a common reducing agent that is abundant within the TME, allowing the detachment of liposome from CTL surface. In vitro results demonstrated that the coupling of liposome to CTL surface was reversible, and the coupling ability could be improved by T cell activation correlated with CD25 receptor expression. More importantly, liposome coupled CTLs could proliferate and cause cytotoxic effect against targeted tumor cells, indicating that the surface attachment did not affect cell proliferation and function. On account of this, another study by Huang et al. showed that polyclonal T cells expressed lymph node-homing receptors incorporated with lipid nanocapsules (NCs) could act as efficient carriers with active targeting ability to lymphoma cells [27]. The lymph node-active targeting ability is crucial because lymphoma is unlike solid tumors, which lack the EPR effect, making the potent therapeutic agent SN-38 display poor pharmacokinetics. In this study, SN-38 was entrapped into NCs containing multilamellar lipid which could covalently bind to free thiols at T cell surface by the formation of a thioether bond. The NCs-functionalized polyclonal T cells could migrate to the lymphoid organs where lymphoma cells resided, which was achieved by organ-specific targeting rather than tumor-specific targeting. In a disseminated lymphoma mice model, this T cell-based DDS reduced tumor burden significantly owing to the enhanced delivery of SN-38 to tumor cells.

In addition to intracellular loading and surface coupling, T cell membrane camouflaged NPs is the third approach for T cell-based vector development. Ma et al. exploited a novel biomimetic nanomaterial based on CAR-T cell membrane-cloaked mesoporous silica NPs (MSNs), aiming to deliver IR780 dye for hepatocellular carcinoma (HCC) treatment [28]. IR780, a photothermal agent, were encapsulated into MSNs to form a biodegradable core. IR780-loaded MSNs were further coated by membrane derived from lentivirus transfected CAR-T cells that could express glypican-3 (GPC3)-specific CARs which enabled CAR-T cells to target HCC tumors by recognizing GPC3 on HCC cells. Both in vitro and in vivo results revealed that GPC3-specific CAR-T cell mem-brane camouflaged IR780-loaded MSNs (CIMs) could selectively accumulate in HCC cells, causing a significant PTT effect for tumor ablation upon 808 nm laser irradiation.

#### 2.2.2. Natural Killer Cells

Similar as T cells, NK cells have also been utilized to create novel cell-based drug delivery platforms in several ways [81]. NK92 cell line is the most extensively characterized type of NK cells and has been approved by FDA for cancer immunotherapy [82]. Siegler et al. constructed drug carriers based on CAR-engineered NK92 cells (CAR.NK cells), whose surface was attached by cross-linked multilamellar liposomal vesicles (cMLVs) containing PTX [29]. CAR.NK cells were generated via retroviral transduction, aiming to increase their homing ability, selectivity, and cytotoxicity towards tumor cells. The cMLVs were functionalized with thiol-reactive maleimide head groups that could covalently conjugate with free thiols highly expressed on NK cell surface, allowing stable attachment of cMLVs to CAR.NK cells surface. An appropriate dose of PTX was encapsulated into cMLVs, which could kill tumor cells and showed no toxicity to CAR.NK cells. According to in vitro experiments, cMLVs were not internalized by NK cells after attachment, and did not cause damages on NK cells, which retained their migratory ability to chemoattractant. CAR.NK cells also released interferon (IFN)-γ; thus, they caused specific cytotoxicity to antigen-expressed cancer cells. In SKOV.CD19 ovarian tumor-bearing mice, the developed DDS CAR.NK.CLV could home to tumor sites and promote PTX accumulation within tumor niches, leading to significant tumor growth inhibition.

The NK cell membrane is also regarded as candidate for NK-cell based drug de-livery. Pitchaimani et al. combined liposomes and NK cells to newly design a kind of liposomal drug vector called NKsome through the infusion of NK cell membrane into liposomes driven by electrostatic interactions [30]. NK92 cells were chosen in this study also, due to their outstanding characteristics, including easy-expansion ex vivo, broad-spectrum targeting capacity, and promoted cytotoxicity. These fusogenic NKsomes could retain the surface receptor proteins of NK92 cells, which allowed NKsomes to mimic properties similar to NK cells, such as tumor homing capacity and immunosurveillance of cancer cells. Furthermore, NK cell membrane-fused NKsomes had positive charged surfaces, which enabled them to escape from lysosomal degradation in target cells due, to the fusion property of NKsomes with the target cell mem-brane. Moreover, this fusion property could realize accurate release of encapsulated drug inside tumor cells. In vitro investigation demonstrated that NKsomes were stable in serum and accumulated significantly in MCF-7 breast cancer cells with wonderful targeting efficiency; therefore, when loaded DOX into NKsomes, the constructed nanocomplexes (DOX@NKsomes) showed superior cytotoxicity to MCF-7 cells without significant immunogenicity. In addition, in vivo results revealed that DOX@NKsomes had longer circulation time and distributed mainly within the TME, which induced a significant inhibitory effect on MCF-7 tumor growth.

### 2.3. Macrophages and Monocytes

Macrophages are a type of phagocyte and can be roughly classified into M1 macrophages (classically activated macrophages) and M2 macrophages (alternatively activated macrophages) [83]. M1 macrophages promote immune response against tumor cells by secretion of inflammatory cytokines with potent antitumor activity such as tumor necrosis factor, while M2 macrophages show an anti-inflammatory function [84]. On this account, a shift of M2 macrophages towards M1 is in favor of tumor sup-pression. Monocytes are mononuclear phagocytes that have an important impact on tumor growth and progression. In addition, monocytes can differentiate into a macrophages or dendritic cells (DCs) under certain conditions [85]. Tumor cells can generate monocyte chemoattractant protein-1 (CCL-2), also known as MCP-1, which is able to recruit monocytes and macrophages [86]. For this reason, monocytes and macrophages have a tumor targeting ability that can be employed for drug delivery.

Macrophages, as a kind of inflammatory cell, have an innate chemotaxis capacity, which means that they can be driven to inflammation by inflammatory factors such as tumor necrosis factor (TNF)-α, interleukin (IL)-10. C-C chemokine receptor type 2 (CCR2) is expressed on macrophages in response to CCL-2, allowing the specific migration of macrophages towards TME. In addition, an essential requirement that enables the feasibility of cell-based drug delivery is that the loaded drug does not induce damage to carrier cells and would not be degraded inside the cell. Hence, in view of these facts, Qiu et al. exploited a “dual-guide” drug delivery vector based on macro-phages for triple-negative breast cancer treatment [31]. It is essential to give chemo-therapy after surgery for triple-negative breast cancer treatment due to the particularly high risk of recurrence. Given that a sustain inflammation occurs in tumor sites after tumor resection, drug-containing liposomes loaded macrophages were developed for tumor targeting achieved by dual guide of tumor and inflammation, aiming to ensure the distinguished targeting ability. Liposomes were PEGylated and modified with octaarginine (R8), a cell penetrating peptide for promoting tumor cell penetration, to load resveratrol (Res) and PTX simultaneously. The obtained liposomes were co-incubated with macrophages to construct the final system PTX/Res-R8-Lip@MP without damage and polarization on macrophages. In vitro results revealed that PTX/Res-R8-Lip@MP could migrate by nanotube formation in response to both inflammation and tumor attraction and enter into tumor cells accompanied by the re-lease of liposomes. The released R8-modified PEGylated liposomes were taken up by tumor cells through a “two-way delivery” mechanism—cell membrane fusion and cell penetrating—which could achieve sustained release of therapeutic agent Res and PTX. Res could block the pro-inflammation pathway and combination of Res and PTX could enhance inhibition of tumor initiating cells, which showed the significant anti-recurrence and anti-stemness effect. More importantly, PTX/Res-R8-Lip@MP efficiently inhibited tumor regrowth in a 4T1 orthotopic mouse model, avoided inflammation, and promoted tumor cell apoptosis. Besides, Guo et al. developed engineered macrophages by anchoring lipopolysaccharides (LPS) on their surfaces to deliver DOX for lung cancer treatment [32]. LPS, a biomolecule presented on outer membrane of bacterial, could induce tumor-associated macrophages towards the M1 phenotype, which was beneficial to improve the anticancer efficiency of the proposed LM-DOX formulation. In orthotopic lung cancer mice model, LM-DOX could migrate to tumor efficiently in response to CCL2, which facilitated the specific delivery of the encapsulated DOX to tumor cells and the production of TNF-α through activating TAMs by LPS anchored on macrophages. Hence, LM-DOX could achieve the release of TNF-α release from TAMs and DOX accumulation, leading to a synergetic inhibitory effect against A549 lung cancer cells.

In addition to the examples mentioned above, Molinaro et al. described an innovative approach for the development of proteolipid vesicles, which were derived from leukosomes for inflammation targeting [87]. This novel leukosome platform was constructed via reconstituting proteins derived from the membrane of J774 macrophages into phospholipids bilayer of lipid NPs, which was based on a newly approach to com-bine traditional top–down and bottom–up methods. In brief, membrane proteins of macrophages were isolated firstly, which then incorporated into the preparation process of thin layer evaporation approach. Following the formation of phospholipids thin film, PBS containing proteins were used to hydrate for constructing leukosomes. In vitro characterizations, such as fluorescence microscopy and flow cytometry, con-firmed that critical proteins of leukocytes membrane were successfully transferred on-to the leukosomes, which enabled leukosomes to possess wonderful targeting ability to inflamed endothelia. More importantly, in an LPS-induced ear inflammation mouse model, leukosomes showed enhanced accumulation in the inflamed sites, and the targeting capacity was demonstrated, related to lymphocyte function-associated antigen 1 (LFA-1) and CD45 molecules. In addition, leukosomes could load hydrophilic, amphiphilic, and hydrophobic therapeutical compounds without affecting their structure, shape, and size. Hence, due to the fact that TME was inflammatory, this exploited biomimetic leukocyte proteolipid vesicles based on macrophage proteins and lipid NPs have great potential in anti-cancer targeted treatment.

In a similar manner, monocytes can also be utilized as drug delivery vectors. Ibarra et al. investigated human monocyte cells (THP-1) and murine monocytes employed for drug delivery to carry conjugated polymer NPs (CPNs) that could be ap-plied as an outstanding photosensitizer (PS) for photodynamic therapy (PDT) [33]. CPNs could be taken up by both human monocyte cells and murine monocytes and showed no toxicity to monocytes without irradiation. Moreover, ex vivo monocyte activation by LPS could improve the uptake efficiency, as well as the tumor penetration ability. In vitro results indicated that CPN-loaded monocytes could home into and penetrate glioblastoma (GBM), spheroids, and unloaded intracellular CPNs when monocytes differentiated into macrophages. CPN-loaded murine monocytes could cross blood brain barrier and sufficiently accumulate in tumor sites with no cargo re-lease in blood circulation due to the monocyte tropism introduced by GBM cells in orthotopic GL261 cells xenograft GBM mice model. In addition, Wang et al. also used the monocytes-based vector to deliver chemotherapeutic drug DOX for GBM treatment [34]. The obtained nano-DOX-loaded monocytes (Nano-DOX-MC) could migrate to tumor sites due to the tropism of monocyte. GBM cells stimulated lysosomal exocytosis of monocytes that led to the DOX unloading, followed by internalization of DOX by GBM cells. In an orthotopic GBMU87 MG cell xenograft mice model, a high tumor targeting delivery and cancer cells damage were both realized.

Since the aforementioned monocyte-based drug delivery platforms need ex vivo preparation, which is labor-intensive and time-consuming, an alternative in vivo internalization strategy has been proposed for monocyte-based drug delivery. Yang et al. demonstrated that chitosan polymeric micelles (COSA) could almost be internalized by monocytes in circulation after being injected to mice and then delivered to tumor sites [35]. The proposed micelles were comprised of chitosan (CO) and stearic acid (SA) that could produce an amidation reaction between amine groups on CO and carboxyl groups on SA. COSA micelles were able to be selectively taken up by monocytes, especially the Ly-6Chi subset monocytes, mainly via mannose receptor-mediated mechanism and secondly by a Dectin 1 receptor-mediated mechanism. Internalization of COSA had very low effect on both the COSA micelles and monocytes, for which the COSA could be stable, and monocytes could preserve their intrinsic tumor-homing ability. Subsequently, COSA-loaded monocytes were recruited to tumor sites and differentiated into macrophages accompanied by the release of cargoes, resulting in CO-SA accumulation within tumor sites. Due to the great potential of micelles for drug loading, this monocyte-based COSA platform showed momentous significance for cancer targeted therapy. In a similar way, Zheng et al. constructed gold-silver nano-rods (AuNRs), incorporating CpG ligands onto their surfaces via Au–S bonds, followed by the wrap of apoptotic bodies (ABs) that were generated by tumor cells under ultra-violet (UV) light irradiation [36]. AuNRs were prepared and functionalized easily through a simple process, while they could achieve significant tumor ablation directly, due to their high photo-to-heat conversion efficiency. The resultant formulations, AuNR-CpG/ABs, were phagocytized by inflammatory Ly-6C+ monocytes with high selectivity since ABs as waste materials of cells could be recognized and engulfed by circulating monocytes. After injected into EL4 tumor-bearing C57/BL6 mice, AuNR-CpG/Abs were driven by monocytes to accumulate in tumor based on inherent tendency toward rapid tumor recruitment of monocytes. CpG could elicit a significant immune response by DC maturation, proinflammatory cytokine secretion and effector T cell activation. Under irradiation, AuNR-CpG/Abs performed a synergistic therapy that consisted of a strong photothermal effect by AuNRs and enhanced immunostimulation by CpG, leading to tumor ablation and metastasis inhibition.

## 3. Erythrocyte-Based Drug Delivery Systems

Erythrocytes are known as red blood cells (RBCs) and are the largest population of blood cells [88]. They have no nucleus, as well as mitochondria, whose basic function is to transport oxygen from the lungs to individual tissues in human body [89]. Physio-logically, erythrocytes are 7–8 μm in size, with biconcave discoid shape which pro-vides a massive surface area. Due to the unique cytoskeleton structure, erythrocytes possess an excellent deformability in favor of crossing blood vessels, contributing to their outstanding mobility [90]. In addition, like other cell types, erythrocytes can generate extracellular vesicles (EVs), containing bioactive molecules such as RNA and re-lease them to achieve biological activities. On the basis of these admirable inherent characteristics, erythrocytes and their EVs have great potential to serve as drug carriers for cancer treatment. Firstly, the abundant source of erythrocytes in blood enables the adequate supply of natural vehicles for drug delivery. Hence, erythrocytes are a kind of easily obtained, biocompatible, and non-immunogenic vectors. Besides, erythrocytes have outstanding mobility with long cycle time and large surface area, as well as a large inside volume, which allows a great deal of drugs to be intracellularly loaded or attached on their surface for long circulation delivery. Despite the merits mentioned above, there are still some obstacles, such as lack of tumor targeting ability and drug leakage from destructed cells, that hinder the utilization of erythrocytes as drug carriers. Therefore, cooperation with nanomaterials in erythrocyte-based DDS is a com-mendable approach to overcome these limitations.

Zhao et al. proposed a two-pronged drug delivery strategy based on polymeric NPs assembled on the erythrocytes surface to load anticancer drug DOX for cancer lung metastasis therapy [37]. The polymeric NPs were formulated from PLGA that was biodegradable and could allow sustained release of loaded drugs. In addition, because the diameter of erythrocyte is larger than the lung capillaries, the erythrocytes-based DDS (Ery-DDS) could meet and response to the high shear stress in lung during the circulation, resulting in the dislodging of drug loaded PLGA core and followed by the release of DOX. According to the in vivo results in a B16F10-Luc melanoma lung metastasis model, this Ery-DDS could achieve lung-specific distribution and controlled release of drugs to metastatic nodules, contributing to the enhancement of metastatic inhibition. The tumor targeting capacity of erythrocyte-based DDS is improved by taking advantage of the intrinsic lung narrow capillaries to cause the high risk of lung metastasis. Apart from this, corporation with magnetic materials is another method to realize targeted delivery. Grifantini at al. embedded an antibody and a kind of super-paramagnetic NPs (spmNPs), containing iron oxide, into erythrocytes [38]. After applying an external magnetic field, the erythrocytes, entrapped with spmNPs, could effectively target colon cancer cells both in vitro and in vivo, promoting accumulation of the co-delivered antibody in tumor cells. Moreover, cargoes loaded into erythrocytes could be protected from degradation in circulation and circulate for a longer time.

In addition to living erythrocytes, erythrocytes membrane-coated DDS is another delivery strategy to develop biomimetic NPs. Erythrocyte membranes can preserve the protein of the membrane and possess most intrinsic properties of erythrocytes, which is capable of prolonging the circulation time of cloaked NPs and endowing them with biocompatibility and non-immunogenicity. Hence, erythrocytes membrane-coated biomimetic DDS is another successful carrier to deliver various therapeutic com-pounds. Similar to erythrocyte-based DDS, the erythrocytes membrane-cloaked delivery platform also lacks direct tumor-targeting capacity. Nevertheless, it was found that CD58 and CD59 molecules expressed on erythrocytes membranes could enhance immune recognition and antigen presentation [91]. Shi et al. fabricated a modified erythrocytes membrane-cloaked delivery vector (DSPE-PEG-Man@EM-NPs) with shell–core structures for antigen pep-tides delivery towards DCs [39]. The outer layer was an erythrocytes membrane, with DSPE-PEG-mannose decoration that could allow the DC targeting ability, and the inner core was a kind of self-assembly NP, based on amphiphilic cathepsin B-responsive dipeptide to covalently link antigenic peptides MAGE and NY-ESO-1. In vitro results demonstrated that this vector could reduce the uptake of NPs by macrophages and achieved targeting delivery based on mannose-modified erythrocytes membrane. DSPE-PEG-Man@EM-NPs could promote the maturation of DCs and activate CD8+ T cells, contributing to killing efficiency in both MCF-7 and MDA-MB-231 breast cancer cells. More crucially, in vivo results based on MDA-MB-231 and MCF-7 cells xenograft mice displayed that DSPE-PEG-Man@EM-NPs could effectively perform the broad-spectrum breast cancer inhibition. In addition to drug delivery, Gao et al. exploited an erythrocytes mimic system to deliver oxygen, similarly to real erythrocytes for cancer radiotherapy promotion [40]. Perfluorocarbon (PFC), an inert chemical with extremely high oxygen solubility, was firstly encapsulated by PLGA, followed by the coating of erythrocytes membrane to construct PFC@PLGA-RBCM NPs. These artificial erythrocyte-based PFC@PLGA-RBCM NPs had much smaller size than native erythrocytes in circulation, resulting in enhancement of extravascular diffusion within the tumor mass, due to the EPR effect. Therefore, a large amount of oxygen was delivered to the hypoxic tumor, which could significantly promote the radiotherapy effect.

**Figure 3:**
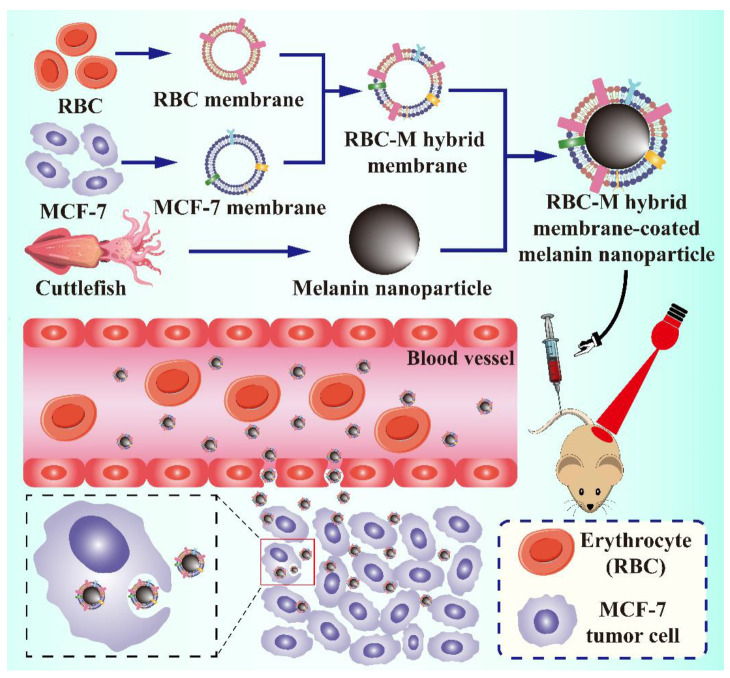
Schematic demonstration of the preparation of erythrocyte-cancer (RBC-M) hybrid membrane-camouflaged melanin nanoparticle (Melanin@RBC-M) for enhanced photothermal therapy in cancer treatment.

Moreover, apart from single erythrocytes with or without decoration, the hybrid membrane system, via fusing the erythrocyte membrane and cancer membrane, has been proposed for cancer therapy in some research. The fused erythrocyte-cancer cell hybrid membrane is capable of performing prolonged blood circulation and homotypic targeting to tumor cells simultaneously, contributing to outstanding anticancer effectiveness in cancer immunotherapy and PTT [92]. For instance, Jiang et al. fused the membrane of erythrocyte with MCF-7 tumor cell membrane to construct an erythrocyte-cancer (RBC-M) hybrid membrane-camouflaged melanin NP (Melanin@RBC-M) to achieve an improved PTT effect [41] (Figure 3). Along with natural biocompatibility and photothermal characteristics from the melanin core, this hybrid NP retained the membrane proteins of both parent erythrocytes and MCF-7 tumor cells, which prolonged its blood circulation and improved its homotypic targetability towards source MCF-7 cells. In vivo results, obtained from MCF-7 tumor-bearing mice exposed to near-infrared laser (NIR) irradiation, showed that when the membrane protein weight ratio of erythrocyte and MCF-7 tumor cells was 1:1, a better PTT efficacy could be obtained, compared with other Melanin@RBC-M in different protein weight ratios and pristine melanin NPs, which was mainly attributed to the optimal balance between the prolonged blood circulation and homotypic targeting.

## 4. Platelet-Based Drug Delivery Systems

**Figure 4:**
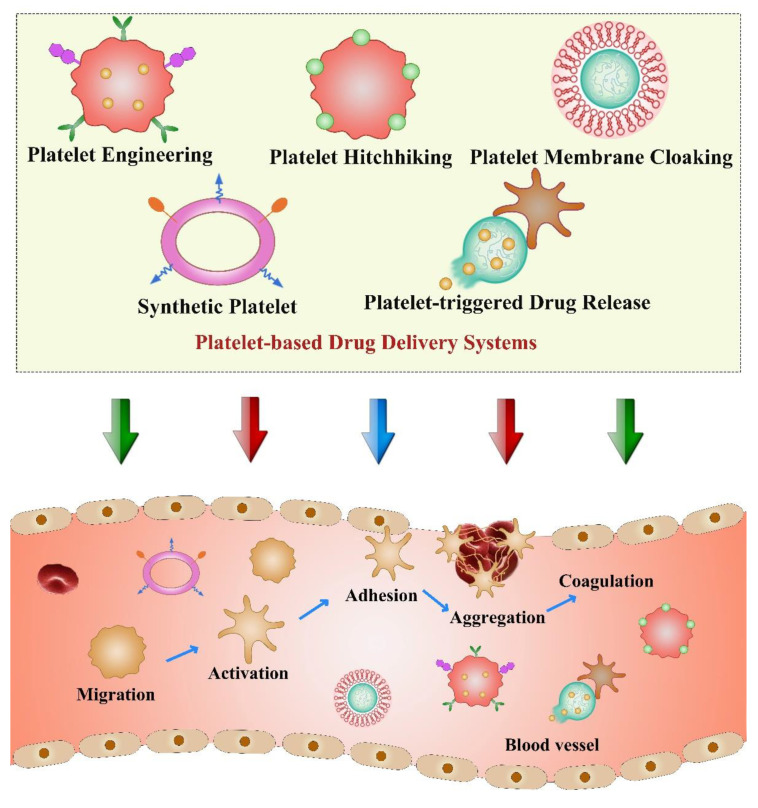
Schematic illustration of various platelet-based drug delivery systems and the steps of hemostasis associated with platelet functions.

Platelets are fragments of cytoplasm that are produced from mature megakaryocytes in bone marrow with the life span of about 8–10 days [93]. Platelets play a central role during vascular injury, wound healing, inflammation, and hemostasis after thrombosis, which are responsible for coagulation, stopping bleeding, and repairing damaged blood vessels [94]. Recently, accumulating research papers have demonstrated the close relationship between platelets and tumor progression. For instance, platelets can contribute to tumor metastasis by promoting tumor angiogenesis and enabling the survival of tumor cells in bloodstream [95]. It is reported that platelets can identify circulating tumor cells (CTCs) and protect them from immune clearance. Moreover, after activation, platelets can change their shapes and release granules including surface molecules and various factors to increase their adhesion with CTCs to form heteroaggregates, causing the phenomenon that is named tumor cell-induced platelet aggregation (TCIPA) [96]. Due to the natural targeting properties of platelets towards tumor cells and vascular disorders, innovative strategies based on platelets were explored for anticancer drug delivery [97]. In addition, platelets possess high drug loading capacity to transport various therapeutic molecules, which is beneficial to their application as biomimetic drug carriers in cancer therapy. Platelets can be directly used as a delivery platform through surface modification or hitchhiking NPs. In addition, construction of platelet-mimicking nanoplatform by coating NPs with plate-let membranes is an alternative method to obtain platelet-derived DDS. Other strategies, such as synthesizing platelets artificially or developing platelet-triggered con-trolled drug release, also attract many researchers’ attention [98] (Figure 4). Therefore, combination of platelets or platelet membranes with nanomaterials will endow whole delivery systems with many distinct advantages, while overcoming their limitations.

CD22 is a cell surface adhesion molecule that regulates B cell activation, which is commonly found in normal B cells and B cell malignant tumors. Xu et al. coupled the anti-CD22 monoclonal antibodies with DOX-loaded platelets to accurately bind to the CD22 receptor on the surface of tumor cells, which precisely delivers DOX for the treatment of B cell lymphoma [42]. In vitro and in vivo results showed that DOX–platelet–CD22 could target tumor tissues, which significantly decreased the tumor volume compared to the control groups. Moreover, the combination of platelets and anti-CD22 monoclonal antibody to deliver DOX could remarkably reduce the cardio-toxicity of DOX, indicating an enhanced therapeutic efficacy with reduced side effects on normal tissues. Therefore, this DOX–platelet–CD22 nanosystem represented a new approach for lymphoma treatment. Immune checkpoint blockade therapy (ICB) has made significant progress in the treatment of various tumors [99]. In light of this, Han et al. engineered platelets with anti-PD-L1 antibodies (aPD-L1) to take the advantage of the excellent inflammatory targeting abilities of platelets for local tumor recurrence and metastasis treatment after photothermal therapy [43]. In their study, PLGA coated indocyanine green (PLGA-ICG) was used as a photothermal agent to result in thermal ablation of tumor tissues, which subsequently caused inflammation and injury to recruit aPD-L1-engineered platelets to the tumor sites, showing great inhibitory effects on residual tumor growth and metastasis by activating the anticancer immune responses. In vitro and in vivo results based on 4T1 breast tumor models showed that this platelet-based nanoplatform possessed outstanding homing potential to ablated tumor sites, which could trigger the release of aPD-L1 upon the platelet activation. In addition, the survival rate for mice receiving aPD-L1-conjugated platelets was significantly higher than control groups, indicating the great therapeutic efficacy of this nanoplatform in breast cancer treatment. Similarly, Wang et al. encapsulated DOX into platelet cytoplasm and modified the surface of DOX-loaded platelets with transferrin (Tf) to establish a Tf-P-DOX DDS for the treatment of melanoma [44]. Transferrin is the main iron-containing protein in plasma, which is responsible for carrying the iron absorbed by the digestive tract and the iron released by the degradation of red blood cells. The transferrin receptor (TfR) is mostly located on the cell surface to mediate the transportation of iron-containing ferritin from outside the cell into the cell. Studies have shown that, compared with nor-mal cells, TfR is highly expressed on the surface of tumor cells. Therefore, the transferrin in the Tf-P-DOX delivery system could act as a targeting ligand to guide the drug-loaded platelets to the tumor site [100]. In vitro results demonstrated that Tf-P-DOX promoted B16F10 cell uptakes and improved their intracellular drug accumulation, exhibiting an increased apoptotic effect on cells compared to free DOX. Moreover, in vivo imaging results, obtained from a melanoma C57BL/6 mouse model, showed that Tf-P-DOX possessed the most NIR fluorescence concentration around the tumor sites, indicating its great targetability towards tumor tissues. In addition, the tumor volume after Tf-P-DOX group treatment was significantly smaller than that of the other groups, indicating its good efficacy to inhibit melanoma growth and progression.

Compared with the direct application of platelets in constructing DDS, platelet membrane-cloaked strategies have received increasing attention in anticancer targeted drug delivery. The integration of platelet membranes with various nanomaterials can retain the distinct properties of platelets and avoid their drawbacks like undesired bio-distribution and unexpected activation-related drug toxicity or efficacy reduction. For instance, Liu et al. developed a pH-responsive, biomimetic nanocarrier based on plate-let membrane coating [45]. The PEOz–platesome–DOX was obtained by coextruding the pH-responsive lipid DSPE-PEOz that encapsulated DOX and platelet membrane nanovesicles (PNVs). The strong affinity between PEOz–platesome–DOX and tumor cells is mainly formed due to the combination of CD62p on the surface of the platelet membrane and the over-expressed CD44 receptor on the surface of tumor cells. In addition, this nanocomposite was pH-sensitive and could release the loaded drugs in the acid microenvironment of lysosomal compartment. In vivo results based on CT26 and 4T1 mice models showed that PEOz–platesome–DOX possessed considerably better antitumor efficacy than other groups, without causing obvious abnormalities on major organs, illustrating its promising application potential in cancer treatment.

Nanocarbon suspension (CNs) is a black suspension liquid that is clinically used to track the draining lymph nodes of gastric cancer. It has good photoacoustic (PA) imaging ability and photothermal conversion characteristics, which are suitable for PA imaging and photothermal therapy [101]. Perfluoropentane (PFP) is a phase change material that mainly used as a contrast media in ultrasound diagnosis. When the liquid PFP is irradiated by the NIR, the optical droplet vaporization (ODV) is activated to form microbubbles, which can release energy when the bubbles burst, increasing the permeability of cells and promoting drug release at the tumor site [102]. PLGA is a de-gradable functional polymer organic compound with good biocompatibility, good encapsulation, and good film-forming properties, which is widely used as a drug carrier in cancer treatment. Li et al. obtained DOX-PFP-CNs@PLGA/PM NPs, named as nano-platelets, by encapsulating CNs, PFP, and DOX in a PLGA skeleton, which was followed by the cloaking of a platelet membrane [46]. This nanoplatelet could target the tumor site under the guide of CD44 on the platelet membrane, and due to the presence of CNs and PFP, it could ablate tumor cells upon laser irradiation, which promoted the release of DOX at the tumor site. In vitro experimental results, based on 4T1 breast cancer cells, indicated that PM enhanced the accumulation of DOX-PFP-CNs@PLGA/PM NPs in tumor cells. Compared with the other groups, the apoptosis rate of the DOX-PFP-CNs@PLGA/PM NPs group was the highest, which proved the good in vitro synergistic therapeutic effect of CNs and DOX in the presence of NIR and PFP. Furthermore, in vivo results, based on 4T1 tumor models, showed that the tumors of mice treated with NPs were completely eliminated, confirming the excellent effect of this nanoplatform on the synergistic treatment of breast cancer. Fe_3_O_4_ NPs are magnetic NPs with wide application in targeted drug delivery, immunoassays, and cancer diagnoses. For example, Jiang et al. loaded sulfasalazine (SAS) into Fe_3_O_4_ magnetic NPs, and then coated them with platelet membrane to obtain Fe_3_O_4_-SAS@PLT, which was used in the cancer treatment through ferroptosis [47]. SAS can inhibit the uptake of cysteine to suppress tumor growth, and then induce ferroptosis. Similarly, Fe_3_O_4_ nanomaterials are ferroptosis inducer. Therefore, the cooperation of Fe_3_O_4_ with SAS can inhibit the Xc-pathway of the glutamate–cystine antiporter system to trigger a ferroptotic cell death. In vitro results showed that Fe_3_O_4_-SAS@PLT NPs camouflaged by platelet membranes had stronger cytotoxicity than Fe_3_O_4_-SAS with enhanced cellular uptake in 4T1 cells. In vivo results based on 4T1-luc metastatic tumor models showed that Fe_3_O_4_-SAS@PLT was concentrated in the liver, spleen, and lung, indicating its outstanding targetability towards metastasis. In addition, when combined with anti-PD-1 treatment, almost no metastatic nodules were found in the lungs compared to the control groups, which showed that this combination therapy effectively inhibited metastatic tumors growth. Black phosphorus (BP) is a non-metallic layered semiconductor with excellent electronic and optical properties. In 2015, Zhang et al. used liquid-phase ultrasound technology to successfully synthesize black phosphorus quantum dots (BPQDs), a kind of zero-dimensional structured nanomaterial of black phosphorus [103]. BPQDs have uniform size, good dispersion, excellent biocompatibility, and low cytotoxicity, which has been widely used in bioimaging and cancer treatment. Hederagenin (HED) is a pentacyclic triterpenoid compound isolated from plants, which has antitumor, antidepressant, antibacterial, and anti-inflammatory effects. Therefore, a nanoplatform called PLT@BPQDs-HED, based on BPQDs, was constructed by Shang et al. They loaded HED on the BPQDs and camouflaged it with a platelet membrane [48]. The particle size of BPQDs is around 10–20 nm, which was difficult for macrophages to swallow. Therefore, BPQDs are more easily distributed to tumor sites. In addition, the acidic environment of TME facilitates the degradation of BPQDs, which can achieve the goal of controlled drug release at the tumor site. In vitro and in vivo results, based on MCF-7 breast cancer models, demonstrated that PLT@BPQDs-HED group had an enhanced cytotoxicity with a better ability to induce autophagy and cell apoptosis than free HED alone. Besides, the tumor volume in the PLT@BPQDs-HED group was significantly reduced with more necrotic cells identified than control groups, indicating its powerful efficacy in breast cancer treatment.

## 5. Stem Cell-Based Drug Delivery Systems

Stem cells are a type of cell with unlimited or immortal self-renewal capacity, which are capable of producing at least one type of highly differentiated progeny cells. According to the classification of developmental stages, they can be divided into two categories: embryonic stem cells (ESCs) and adult stem cells (ASCs) [21]. ECSs have developmental totipotency and can, theoretically, induce differentiation into all kinds of cells in the body [104]. ASCs refer to the undifferentiated cells that exist in a differentiated tissue. Under certain conditions, these cells either produce new stem cells, or differentiate according to certain procedures to form new functional cells. Therefore, tissues and organs can maintain a dynamic balance of growth and decline [19]. The use of stem cells as cellular vehicles to carry drug-loaded NPs was demonstrated as a very promising strategy for antitumor targeted therapy [105]. 

**Figure 5:**
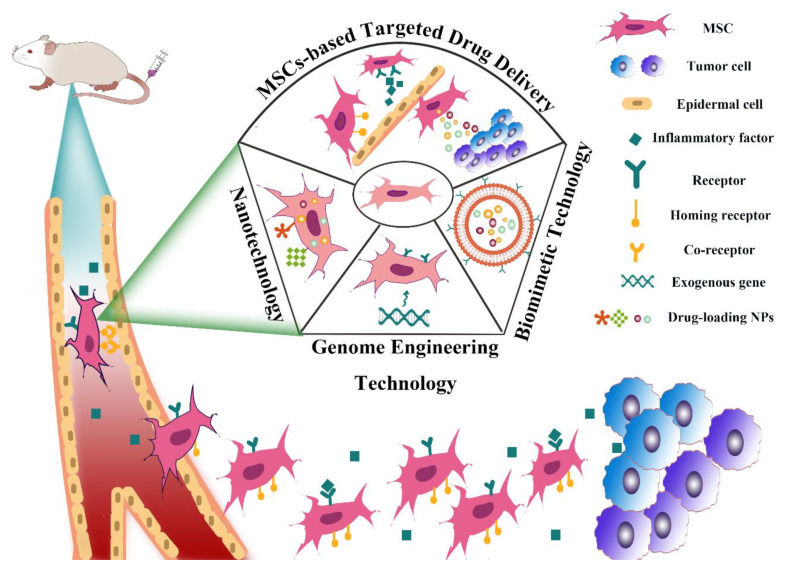
An overview of MSC-based drug delivery systems and the process of their homing to tumor sites.

Mesenchymal stem cells (MSCs), adipose-derived stem cells (ADSCs), and neural stem cells (NSCs) all belong to ASCs. Because of their tumor tropism, they can be used as drug delivery vehicles in cancer treatment [55,106]. In particular, MSCs belong to a kind of pluripotent stem cells in the mesoderm, which have a strong proliferation ability with multi-directional differentiation potential. MSCs can continuously migrate from the original tissue site to the new tissue site; thereby, they participate in the renewal and repair of tissues and organs under physiological or pathological conditions, which can maintain the morphological integrity and functional stability of the body. In addition, a large number of studies have found that when the body is ischemic, hypoxic, and injured, the internal or exogenous MSCs have the characteristics of dominant distribution to the injured site, indicating their strong migration ability. Therefore, owing to their tumor tropism towards a variety of tumor tissues, MSCs have been widely studied as drug delivery vehicles in cancer treatment [107,108,109]. The homing mechanism of MSCs is not fully clear. In general, different microenvironments secrete different signal molecules, such as chemokines, growth factors and adhesion factors [110,111,112]. These signal molecules bind to the corresponding receptors on the MSCs membrane to drive their homing behavior together. In addition, the source of stem cells also has influences on the homing capacity of MSCs, which may be resulted from the different receptors on the cell surface. For example, compared with bone mar-row-derived MSCs, placental-derived MSCs have higher expansion and engraftment activity [113]. Moreover, Senthilkumar et al. showed that the homing ability of dental pulp-derived MSCs to damaged neurons was significantly higher than that of bone marrow-derived MSCs, which may be due to the overexpression of homing factors in dental pulp-derived MSCs compared with bone marrow-derived MSCs [114]. Moreover, MSCs have other advantages as cellular vehicles. For example, MSCs come from a wide range of sources and can be easily isolated and expanded in vitro [50]. MSCs have no neurotoxicity with lower immunogenicity, which can be genetically modified to serve as a vehicle for cancer gene therapy with little or no impact on their biology [115]. Moreover, MSCs can be engineered to express pro-apoptotic or anti-angiogenetic proteins for the treatment of several tumor types [116,117]. Altogether, these biological properties of MSCs make them a new generation of drug delivery vehicles for cancer therapy (Figure 5).

In recent years, various functional or drug-loaded NPs have been used to intro-duce new functions into MSCs and enrich the therapeutic potential of MSCs-based DDS. Several methods have been adopted for MSCs to carry NPs as follows: (1) Incubating MSCs and NPs together to load NPs into the MSCs through endocytosis. The cell chamber of MSCs enables the storage of NPs in their cytoplasm [118]. (2) NPs can be attached to the surface of MSCs either by covalent conjugation strategies or physical associations mediated via electrostatic and hydrophobic interactions [113]. (3) Application of MSCs’ membrane as drug carriers to combine the biomimetic properties of MSCs with the enhanced functionality of synthesized nanomaterials. Because the homing ability of MSCs is largely due to the functional molecules expressed on cell membranes, such as selectin, integrin, C-C chemokine receptors, growth factors, and adhesion molecules; therefore, cloaking the membrane of MSCs around NPs will retain their merits to a great extent [105]. (4) MSC can transfect genes to express antitumor proteins. Genome engineering technology is a method of modifying cells by introducing foreign genes with specific sequences into host cells through different gene vectors. Thus, a variety of functional genes can be used to improve the targeting ability of MSCs-based DDS through this novel technology [52]. The genetic engineering of MSCs is usually accomplished by using viral or non-viral gene vectors. At present, the most studied viral gene vectors are retroviruses, lentiviruses, and adenoviruses. However, these methods cannot avoid the potential risks of viral transduction. Therefore, non-viral vectors, including various lipids, polymers, and inorganic NPs, are alternative carriers for gene delivery [119,120].

The combination of MSCs-based DDS with nanomaterials can improve the targetability of the whole DDS due to the unique property of MSCs. To this aim, Kim et al. developed a systematic strategy for the elimination of lung tumors using nanodrug-conjugated MSCs [49]. In their study, carbon nanotube (CNT) was mixed with anti-CD90 and DOX to obtain the covalent anti-CD90–DOX–CNT conjugate. Then, the nanodrug was conjugated on MSCs to form the MSC–nanodrug conjugation. In vitro results, based on lung cancer cells, showed that the homing ability of MSC–nanodrug conjugations to lung cancer cells was similar to that of unmodified MSCs, which could efficiently promote lung cancer cell apoptosis. In vivo results, based on lung tumor-bearing mice, demonstrated that the luciferase intensity of lung tumors decreased significantly, and the lung tumor was eliminated after treatment. In general, the nanodrug conjugated on MSCs successfully destroyed lung cancer cells by using the homing ability of MSCs to the lung tumor. Moreover, Takayama et al. encapsulated the anticancer agent DOX into liposomes to form DOX-loaded liposomes (DOX–Lips). Then, murine mesenchymal stem cell line C3H10T1/2 was modified with DOX–Lips using the avidin–biotin complex (ABC) method to carry drugs for transportation. The anti-tumor effect of the DOX–Lip–C3H10T1/2 cells were examined using a murine colon adenocarcinoma cell line by co-culturing and tracking DOX–Lips on the surface of C3H10T1/2 cells. In vitro experimental results showed that, compared with DOX–Lips alone, DOX–Lip–modified C3H10T1/2 cells significantly inhibited the proliferation of the mouse colon adenocarcinoma cell line Colon26/Fluc that expresses firefly luciferase. Notably, in vivo results demonstrated that DOX–Lip–modified C3H10T1/2 cells suppressed tumor growth in subcutaneous tumor-bearing mice and in the lung metastasis model. In summary, all these results indicated that the application of DOX–Lip–modified MSCs was an effective approach to enhancing the intercellular de-livery of DOX for targeted antitumor therapy [50].

Paris et al. showed that gene transfection of MSCs with plasmids could be successfully attained using a polyethylenimine (PEI) coating of MSNs. Polycation-coated ultrasound-responsive MSNs (UR-NPs) were obtained by encapsulating UR-NPs with PEI. Then, the plasmids that contained a suicide fusion gene composed of the sequences of cytosine deaminase (CD) and uracil phosphoribosyl transferase (UPRT) were combined with the PEI coating and transfected into MSCs. Then, the gene transfected MSCs were co-cultured with NMU rat mammary cancer cells. An Alamar Blue assay showed a significant decrease of NMU cell viability and apoptosis–necrosis analysis (by flow cytometry) showed that the number of early apoptotic NMU cells increased significantly when employing this therapeutic strategy, compared with the other control conditions. In summary, gene transfection of MSCs with plasmids encoding cyto-sine deaminase and uracil phosphoribosyl transferase suicide genes was an efficient and hypotoxic method for MSCs-based targeted therapy of tumor [51]. An entirely novel bioinspired approach was adapted by Chen et al. for the prevention of pulmonary metastasis of melanoma [52]. In their study, a TRAIL-decorated nanovector, composed of reconstituted high-density lipoproteins (rHDL), was developed to target MSCs that overexpress scavenger receptor B type I (SR-BI). The pDNA that encoded the genetic expression of TRAIL was used as a vector to anchor the pulmonary MSCs. For the construction of this bioinspired nanovector, pDNA was electrostatically con-jugated to a cationic copolymer, composed of PEI and lauric acid. The developed NPs had uniform particle size and good stability in plasma. In vitro results based on B16F10 melanoma cells showed that TRAIL-expressing MSCs, transfected with rHDL, could induce apoptosis of B16F10 cells. In vivo results, based on B16F10-tumor bearing mice, demonstrated that, compared with the control group, rHDL-transfected TRAIL-expressing MSCs had a significant inhibitory effect on the growth of pulmonary metastasis tumors. Taken together, TRAIL-expressing MSCs engineered by the rHDL nanovector was an efficient and hypotoxic method for stem cell-based pulmonary melanoma metastasis-targeting therapy, which not only prevented side effects, but also inhibited metastasis of the melanoma cells.

## 6. Adipocyte-Based Drug Delivery Systems

**Figure 6:**
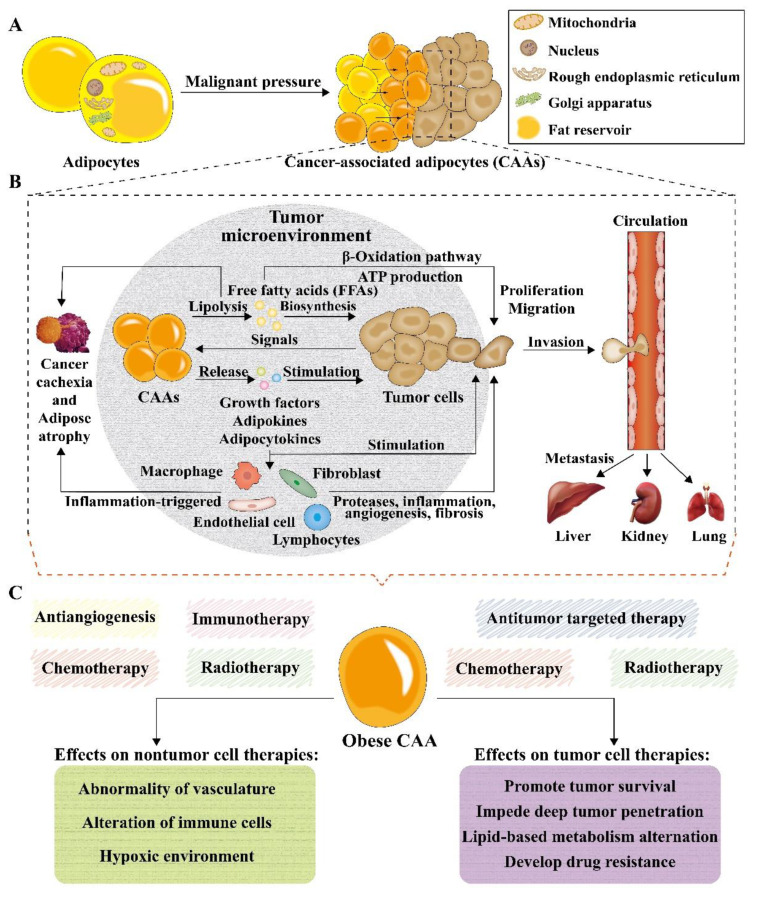
Schematic illustration of the multifunctional roles of adipocytes in cancer development and treatment. (**A**) The composition of adipocytes and their switch into cancer-associated adipocytes (CAAs) upon malignant pressure. (**B**) Mechanisms and interactions of CAAs in tumor progression, metastasis and cachexia. (**C**) Effects of obese CAAs on nontumor cell therapies and tumor cell therapies.

Adipocytes, the main components of adipose tissues, were firstly considered as simple depots to store and provide energy. In recent decades, adipocytes have evolved into fully functioning endocrine and paracrine cells, due to the foundation of leptin, which can regulate systemic energy and metabolic homeostasis [121,122]. Moreover, many adipokines and cytokines were found to be involved in cancer growth and metastasis [121]. Currently, adipocytes can be divided into four subtypes: white adipocytes, brown adipocytes, beige adipocytes, and pink adipocytes [123,124]. It was re-ported that most cancer types are associated with white adipocytes, and that cancer cells can produce signaling molecules to drive normal adipocytes into cancer-associated adipocytes (CAAs) (Figure 6A) [125]. Adipocytes that are found to neighbor the malignant cells are regarded as CAAs, including intratumoral and peritumoral adipocytes. Specifically, malignant cells in the TME make direct contact with intratumoral and peritumoral adipocytes through a range of growth factors and adipokines, promoting the growth, survival, and migration of tumor cells. The production of growth factors and cytokines can also recruit fibroblasts, endothelial cells, and macrophages to TME, causing inflammation-triggered cancer cachexia. Free fatty ac-ids (FFAs) released by lipolysis of adipocytes can also promote the growth, proliferation, and migration of cancer cells. After proliferating and migrating around the tumor site, cancer cells usually invade and circulate in blood vessels and then metastasize to the liver, kidney, and lung (Figure 6B) [126]. Targeted cancer therapies can be classified into two categories: targeting cancer cells and targeting cellular and molecular components of the TME. Chemotherapy and radiotherapy have been widely used to target cancer cells. On the other hand, antiangiogenesis and immunotherapy can prevent the abnormal angiogenesis of tumor cells or regulate the immune environment of the TME, which also inhibits tumor growth [127]. However, obese CAAs can influence the therapeutic effect on both tumor cell therapies and nontumor therapies, which impede deep tumor penetration, change lipid-based metabolism, develop drug resistance of tumor cells, cause abnormal vasculature, alter the function of immune cells, and result in hypoxic TMEs, promoting the survival of tumor cells in anticancer therapy (Figure 6C) [126].

In recent years, the close interactions between tumor cells and adipocytes make adipocytes ideal vectors in anticancer targeted drug delivery. Similar to other cell-based delivery systems, the adipocyte-based delivery system also possesses high biocompatibility, long circulation time in vivo after systemic injection, and great tropism towards tumors and inflammatory sites. Moreover, hydrophobic drugs can be easily encapsulated in adipocytes because of their lipid content. In order to support the metabolism of tumor cells, lipid droplets can accumulate during the lipolysis process triggered by tumor cells, which can be used as a “Trojan horse” strategy for local and sustained delivery of different drugs at the tumor sites. Moreover, the isolation and characterization of adipocytes are specific and economical, which is beneficial in the construction of a DDS [128]. Altogether, adipocytes have been leveraged as a drug delivery depot to achieve local and gradually release of chemotherapeutics for cancer therapy.

For instance, Wen et al. exploited the lipid metabolism to engineer adipocytes that served as a depot to deliver anticancer therapeutics at the tumor sites [53]. In their study, adipocytes entrapped with a reactive oxygen species (ROS)-responsive DOX prodrug (pDOX) and rumenic acid (RA) could cause tumor cells damage through the activation of lipid metabolic pathway mediated by fatty acid-binding protein 4 (FABP4). Moreover, a downregulated expression of PD-L1 could be observed in tumor cells, which was advantageous to CD4^+^ and CD8^+^ T cell-mediated immune responses. Another example by Huang et al. used adipose-derived stem cells (ADSCs) to carry nanotherapeutics for selective homing to brain tumors [54]. Superparamagnetic iron oxide NPs (SPIONs) were entrapped into the PTX-loaded NPs that could subsequently be internalized by ADSCs to achieve a stimulus-responsive effect for integrated hyperthermia and chemotherapy against tumors. Upon chemotactic recruitment of the ADSC-based nanotherapeutics to tumor sites, hyperthermia, and drug release could be initiated by applying high frequency magnetic field (HFMF). In vitro and in vivo results demonstrated the selective delivery of ADSCs-based delivery system, which significantly prolonged the survival time of brain glioma-bearing mice. Furthermore, Aoki et al. designed an ADSC-based DDS which loaded PLGA that conjugated with pirarubicin [55]. Pir-PLGA NP-loaded ADSCs inhibited the growth of tumor cells through the recruitment of ADSCs to tumor site and the dual effect of the released pirarubicin, which had an inhibitory effect on DNA replication and could secrete anti-pancreatic cancer factors. In vitro and in vivo results showed that Pir-PLGA NP-loaded ADSCs could migrate into the inside of the tumor and release pirarubicin gradually, inhibiting human pancreatic cancer cells (KP1N) proliferation and inducing their apoptosis. Moreover, Pir-PLGA NP-loaded ADSCs could inhibit tumor growth at a very low dose and cause minimal side effects, compared with the treatment of pirarubicin alone.

Except for adipocytes, the lipid droplet, an important organelle of adipocytes, was regarded as another efficient drug vehicle for cancer therapy. Since cancer cells need lipid to support their proliferation, invasion, and metastasis, and lipid drop-lets are natural storage for lipid, lipid droplet-coated chemotherapeutics can be selectively delivered to tumor sites as well. Liang et al. showed that pyrolipid, a lipid-based photosensitizer coated with lipid droplets (Pyrolipid@LDs), could significantly inhibit the growth of tumor cells by generation of ROS, lipid peroxidation, and endoplasmic reticulum (ER) stress [56]. In vitro cytotoxicity showed that Pyrolipid@LDs exhibited a more obvious anticancer effect and had less dependence on the normoxic condition, compared with pure pyrolipid, because of the existence of lipid droplets. Also, in vivo anti-cancer activity, conducted in a SKOV3-tumor mouse model, demonstrated that Pyrolipid@LDs with laser could induce the withdrawal of tumor cells. As a result, Pyrolipid@LDs-mediated PDT showed that lipid droplets not only served as the storage for lipid but also participated in the metabolism of tumor cells and could improve anti-tumor efficacy when loaded with chemotherapeutics. In addition, compared to adipocytes-based delivery systems, adipocyte-derived anticancer lipid droplets have less composition and are easier to freeze-dry into powder, showing great potential in anticancer drug delivery.

## 7. Conclusions and Outlook

Nanomaterial-involved cell-based DDSs have opened a new horizon for cancer treatment. Delivery of therapeutics using nanomaterial-mediated cell-based carriers is a very exciting and rapidly developing research area with great clinical benefits. The variety of cell types and the diversity of cell surface conjugation chemistries provide more possibilities for exploring a wide range of NP-encapsulated or nanomaterial-decorated DDSs with excellent targetability towards tumor tissue or other specific sites of interest. Herein, we summarize the most commonly adopted cellular delivery system, which is derived from various cell types including different leucocytes, erythrocytes, platelets, stem cells, and adipocytes. Moreover, a number of novel nanomaterials that come from liposome, polymer, protein, EVs, black phosphorus, gold, silica, iron oxide and carbon nanotubes have been utilized to endow these cell-based DDSs with more attractive properties, owing to their unique physiochemical features. Therefore, these constructed endogenous drug carriers holds a lot of advantages over conventional carries such as good biocompatibility, long circulation time, non-immunogenicity and natural targeting potential.

Although cell-based DDSs have made huge progress in improving the therapeutic performance in cancer treatment, there are still many challenges that impede its clinical practice. One of the biggest difficulties that hinders its development path is that of how to retain the original properties of these living cells after isolating them in vitro to load therapeutic molecules either through internalization or cell surface modification. Obviously, the loaded drugs or NPs should not be toxic to their cell carriers and will not cause cellular alternations after they are carried. In light of this, more work should be done to maintain the balance between cell carriers and NPs to achieve the best therapeutic efficacy. In addition, establishment of a standard protocol in terms of large-scale production is also a challenge that limits the clinical translation of cell-based therapies. Another unavoidable issue is that therapies based on living cells or cell membrane-cloaking strategies can be very expensive because of their highly personalized properties. The high costs mainly stem from the utilization of patients’ own cells that are individually prepared for each patient. Therefore, exploring novel strategies that are able to attach NPs to cells directly in vivo might potentially address this problem. Finally, as the mechanisms of some cellular interactions, as well as the development and metastasis of some cancer types, are still unclear, cell-based nanodrug carriers may also exacerbate their pathological conditions in turn. For in-stance, platelets are highly reactive blood elements, which means that plate-let-mediated drug delivery could cause the risk of unexpected aggregation or the disturbance of platelet function. For stem cells, their genetic risk remains controversial, which requires much further work. For leucocytes, their preparation process and quality control tend to be more difficult. Leucocyte-based DDSs may face the challenges of overloading the RES and immune system, which might cause the risk of affecting host immune defenses. Moreover, if there are too many leucocyte-based carriers in body, the immune system can be activated to release pathological mediators such as cytokines and ROS, which may contribute to aggravated inflammation.

Despite the existing challenges mentioned above, it is certain that cell-based drug delivery strategies, in cooperation with nanomaterials, have enormous potential to revolutionize current cancer treatment. Advancement in developing innovative nanomaterials may open new doors to design more rational DDSs. Although significant work needs to be done, we are confident that the future of this novel anticancer strategy is promising.

## Figures and Tables

**Table 1:** Overview of the combination of nanomaterials in cell-based drug delivery systems for cancer treatment.

Type of Living Cells/Cell Membranes	Combined Nanomaterials/Applied Targeting Molecules	Advantages	Therapeutic Agents	Tumor Model	Therapeutic Performance	Refs.
Neutrophil membrane	PLGA	Achieve efficient tumor targeting, prolong circulation time and promote cellular internalization	Paclitaxel	Human ovarian adenocarcinoma	Inhibit tumor growth and prolong the survival rate	[24]
Neutrophil	PEG-b-PLGA and bacteria-secreted outer membrane vesicles	Improve tumor targeting, combine chemotherapy with PTT	Cisplatin	Murine breast cancer	Completely eradicate tumors	[25]
T cell	Gold nanospheres	Improve tumor targeting through the recognition of tumor-associated antigens	AuNPs	Human lymphoma	Achieve specific tumor AuNPs accumulation	[26]
	Lipid nanocapsules	Achieve lymphoid organ-specific targeting	SN-38	Murine lymphoma	Reduce tumor burden significantly	[27]
CAR T cell membrane	Mesoporous silica	Improve tumor targeting, prolong circulation time	IR780	Human hepatocellular carcinoma	Possess significant photothermal antitumor effect and tumor imaging	[28]
CAR NK cell	Cross-linked multilamellar liposomal vesicles	Improve tumor targeting	Paclitaxel	Human ovarian cancer	Inhibit tumor growth	[29]
NK cell membrane	Liposome	Improve tumor targeting, prolong circulation time	Doxorubicin	Human breast cancer	Inhibit tumor growth	[30]
Macrophage	Liposome	Improve tumor targeting, promote cellular internalization, recruited to tumor sites by CCL-2	Resveratrol and Paclitaxel	Murine breast cancer	Inhibit tumor recurrence	[31]
Engineered macrophage	Lipopolysaccharide	Improve tumor targeting, induce secretion of TNF-α	Doxorubicin	Human lung cancer	Increase the inhibitory effects on tumor growth and metastasis	[32]
Monocyte	Polymer	Improve tumor targeting	Conjugated polymer NPs (CPNs)	Murine glioblastoma	Efficiently deliver CPNs into glioblastoma sites and improve PDT effect	[33]
	N/A	Cross endothelial barriers and improve tumor targeting	Doxorubicin	Human glioblastoma	Induce cancer cell damage	[34]
	Chitosan polymeric micelles	Improve tumor targeting	N/A	Murine breast cancer	Increase NPs accumulation within tumor sites, enhance antitumor efficacy	[35]
	Gold-silver nanorods (AuNRs)	Improve tumor targeting, promote immunostimulation	AuNRs and CpG	Murine lymphoma	Ablate primary tumors and elicit a potent immunity to prevent tumors from metastasis and recurrence	[36]
Erythrocyte	PLGA	Enable lung physiology-assisted shear-responsive targeted delivery	Doxorubicin	Murine melanoma	Inhibit tumor growth and metastasis	[37]
	Iron oxide-based super-paramagnetic NPs	Improve tumor targeting, prolong circulation time	Monoclonal antibody mAb198.3	Human colon-rectal cancer	Inhibit tumor growth	[38]
Erythrocyte membrane	DSPE-PEG-mannose	Improve tumor targeting, possess outstanding mobility	Antigen peptides self-assembled NPs	Human breast cancer	Promote DC maturation and CTL activation, achieving broad-spectrum breast cancer inhibition	[39]
	PLGA	Prolong circulation time, efficiently load and deliver oxygen to hypoxic tumor	Perfluorocarbon	Murine breast cancer	Promote cancer radiotherapy	[40]
Erythrocyte-cancer cell hybrid membrane	N/A	Prolong blood circulation, improve targetability and PTT effect	Melanin nanoparticle	Human breast cancer	Inhibit tumor growth	[41]
Platelet	Anti-CD22 antibody	Prolong circulation time and achieve previse delivery of DOX to tumor cells	Doxorubicin	Human lymphoma	Inhibit tumor growth and attenuate cardiotoxicity of DOX	[42]
	Anti-PD-L1 antibody	Excellent inflammatory targeting ability	N/A	Murine breast cancer	Reduce residual tumor growth and metastasis	[43]
	Transferrin	Effectively target melanoma	Doxorubicin	Murine melanoma	Reduce melanoma cell growth and inhibit tumor progression	[44]
Platelet membrane	DSPE-PEOz liposome	Enhance tumor affinity and achieve selective drug release in acidic microenvironment	Doxorubicin	Murine colon cancer, breast cancer and pancreatic carcinoma	Inhibit tumor growth	[45]
	PLGA	Achieve active targeting and immune evasion abilities	Doxorubicin	Murine breast cancer	Eliminate tumor completely and enhance multimodal imaging	[46]
	Fe_3_O_4_ NPs	Promote targetability to tumor metastasis	Sulfasalazine	Murine breast cancer	Inhibit the metastatic tumor growth	[47]
	BPQDs	Improve drug loading efficiency, enhance biocompatibility and targetability	Hederagenin	Human breast cancer	Inhabit tumor growth and decrease the side effects of myelosuppression	[48]
Mesenchymal stem cell	Carbon nanotubes	Improve tumor homing ability	Doxorubicin	Human lung cancer	Promote lung cancer cell apoptosis and eliminate lung tumor after treatment	[49]
	Liposome	Enhance the intercellular delivery of DOX and improve tumor targeting ability	Doxorubicin	Murine colon adenocarcinoma	Significantly inhibit tumor proliferation, Suppress primary tumor growth and lung metastasis	[50]
Genome engineered mesenchymal stem cells	PEI-coated MSNs	Increase tumor homing ability, reduce undesired side effects of anticancer treatment	A suicide fusion gene and uracil phosphoribosyl transferase	murine NMU mammary tumor	Induce NMU cancer cells death	[51]
	Reconstituted high-density lipoprotein	Increase therapeutic efficiency and tumor targetability	pDNA encoding TRAIL	Murine melanoma	Induce cancer cell apoptosis, inhibit pulmonary metastasis tumor growth	[52]
Adipocyte	N/A	Achieve local and sustained release of chemotherapeutics within the TME	Rumenic acid and doxorubicin prodrug	Murine melanoma	Promote antitumor efficacy, downregulate of PD-L1 expression	[53]
Adipose-derived stem cell	Superparamagnetic iron oxide NPs	Improve selective delivery	Paclitaxel	Murine brain tumor	Enhance therapeutic efficacy and prolong survival time	[54]
	PLGA	Achieve sustained drug release and increase tumor targeting ability	Pirarubicin	Human pancreatic cancer	Inhibit tumor growth, induce the apoptosis of tumor cells, cause minimal side effects	[55]
Lipid droplet	N/A	Promote anticancer therapy through metabolic intervention	Pyrolipid	Human ovarian cancer	Inhibit tumor growth	[56]

## Abbreviations

ADSC	Adipose-derived stem cell
B cell	B lymphocyte
BP	Black phosphorus
CAA	Cancer-associated adipocyte
CAR	Chimeric antigen receptors
CTC	Circulating tumor cell
CTL	Cytotoxic T lymphocyte
DC	Dendritic cell
DDS	Drug delivery system
EVs	Extracellular vesicles
EPR	Enhanced permeability and retention
GBM	Glioblastoma
LPS	Lipopolysaccharides
MSC	Mesenchymal stem cell
NET	Neutrophil extracellular traps
NIR	Near-infrared laser
NK cell	Natural killer cell
NPs	Nanoparticles
PDT	Photodynamic therapy
PEI	Polyethylenimine
PLGA	Poly (lactic-co-glycolic acid)
PTT	Photothermal therapy
PTX	Paclitaxel
RES	Reticuloendothelial system
rHDL	Reconstituted high-density lipoprotein
T cell	T lymphocyte
TME	Tumor microenvironment
ROS	Reactive oxygen species
